# Science Mapping Analysis of COVID-19 Articles Published in Dental Journals

**DOI:** 10.3390/ijerph18042110

**Published:** 2021-02-22

**Authors:** Parisa Soltani, Kimia Baghaei, Kioumars Tavakoli Tafti, Gianrico Spagnuolo

**Affiliations:** 1 Department of Oral and Maxillofacial Radiology, Dental Implants Research Center, Dental Research Institute, School of Dentistry, Isfahan University of Medical Sciences, Isfahan 81746, Iran; p.soltani@dnt.mui.ac.ir; 2 Dental Students’ Research Committee, School of Dentistry, Isfahan University of Medical Sciences, Isfahan 81746, Iran; 3 Department of Neurosciences, Reproductive and Odontostomatological Sciences, University of Naples “Federico II”, 80131 Naples, Italy; gspagnuo@unina.it

**Keywords:** COVID-19, SARS-COV-2, dentistry, dental articles, PubMed

## Abstract

The Coronavirus disease 2019 (COVID-19) pandemic is an ongoing global health crisis with unmatched outcomes and effects. This pandemic has caused an infodemic of article publication in scientific journals. Dental journals have been active in the publication of COVID-19 related articles from the beginning of the pandemic. In this cross-sectional survey, we present an analysis of the scientific output of dental journals on COVID-19. The PubMed COVID-19 database was searched with the “Dental Journals” filter. Data including journal name, country, month of publication and number of citations were recorded. Science mapping analysis of the most used keywords was also performed. The search retrieved a total of 659 articles, of which 28 were excluded. Oral Diseases has published the most COVID-19 articles (15.1%), followed by the British Journal of Oral and Maxillofacial Surgery (8.6%) and the Journal of Dental Education (7.9%). Most of the articles were from researchers from the United States (168), United Kingdom (120) and Brazil (83). The number of citations of the published articles ranged from 0 to 406, with most articles (64.2%) having no citations. Science Mapping analysis revealed that the most used keywords were coronavirus infections, pandemics and humans. The dental community has been active in the publication of COVID-19 articles from the beginning of the pandemic. The papers published by dental journals explore issues such as the management of clinical practices during the outbreak, infection control in the dental setting, signs and symptoms of COVID-19 affecting the oral cavity, and the impact of the COVID-19 pandemic on educational and clinical programs.

## 1. Introduction

The Coronavirus disease 2019 (COVID-19) pandemic is a significant phenomenon in the modern era, leaving its effects on nearly everything. The first cases were reported in China in late 2019, creating a pandemic in early 2020. The total number of cases as of 29 December 2020 exceeded 81 million worldwide [1]. At the beginning, there were many uncertainties about the disease and the virus that causes it, severe acute respiratory syndrome coronavirus 2 (SARS-CoV-2). However, the scientific community has been active in publishing articles investigating different aspects of the disease, including epidemiological features, effective treatment options, signs and symptoms, and how it has affected different operations and communities. Thousands of articles related to COVID-19 and severe acute respiratory syndrome coronavirus 2 SARS-CoV-2 have been published in different scientific journals, giving rise to a unique infodemic. This may be a reflection of the magnitude of the crisis that the global community is currently facing as a result of the COVID-19 outbreak. The output of landmark COVID-19 studies was used by policymakers and clinicians in the battle against the outbreak. The findings of meticulously performed studies can be useful in preventing disease transmission, finding appropriate treatment options for involved patients, or developing effective vaccines [2]. Even with the development and approval of several effective vaccines in early December, COVID-19 seems to remain a significant crisis with considerable long-lasting impacts.

Dentistry as a biomedical field has contributed to the generation and dissemination of knowledge about COVID-19 and SARS-CoV-2 on topics such as salivary diagnostics and the manifestations of COVID-19 in the oral cavity. In addition, one of the most important transmission routes of COVID-19 is through virus-containing aerosols, making dental procedures high-risk operations during the pandemic [3]. Moreover, the effect of the ongoing pandemic on dental professionals and dental education is another topic worth scientific acknowledgement [4]. Additionally, in many countries, dental professionals were required to serve as frontline hospital staff to reduce the burden of the outbreak and deal with the high number of hospitalized patients. Therefore, dental journals are regarded as an important source of information on the dentistry-related implications of COVID-19 and SARS-CoV-2 [5].

While the literature concerning coronaviruses in general date back to the 1960s, the research output about SARS-CoV-2 has been provided in 2019 and 2020 [6]. To the best of our knowledge, the COVID-19-related output of dental journals has not been scientifically analyzed yet. This analysis can provide a general outlook on published articles and their associated features. Therefore, in this article, we aim to present a science mapping analysis of the research output of dental journals on COVID-19.

## 2. Materials and Methods

In this cross-sectional survey, the special COVID-19 database of PubMed was searched (https://pubmed.ncbi.nlm.nih.gov/?term=covid-19, accessed on 3 December 2020) with applying the “Dental Journals” filter. Data including titles, authors, countries, journal names, month of publication and number of PubMed citations were recorded. Exclusion criteria were languages other than English, errata and correction papers, duplicated records and briefing of other articles. The data were imported to a Google spreadsheet (Google, Mountain View, CA, USA) and analyzed using Microsoft Excel version 2010 (Microsoft, Redmond, WA, USA).

Additionally, the PubMed search output was imported into VOSviewer (http://www.vosviewer.com/, accessed on 1 December 2020. Leiden University’s Center for Science and Technology Studies, Leiden, The Netherlands). Medical subject headings (MeSH) keywords were used for science mapping through co-occurrence network analysis. Network visualization was applied with the minimum number of occurrence of the keywords set at 5.

## 3. Results

**Figure 1:**
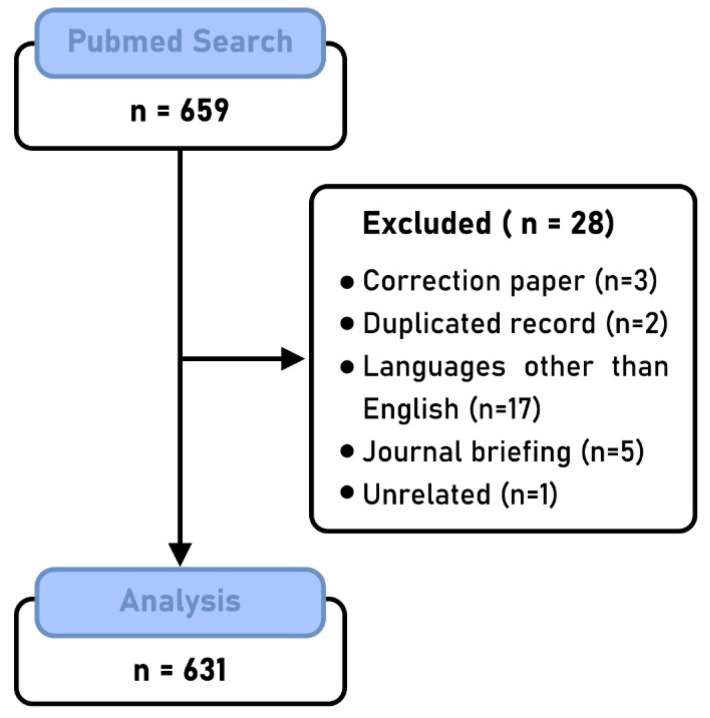
Flow diagram of the articles analyzed in the study.

**Figure 2:**
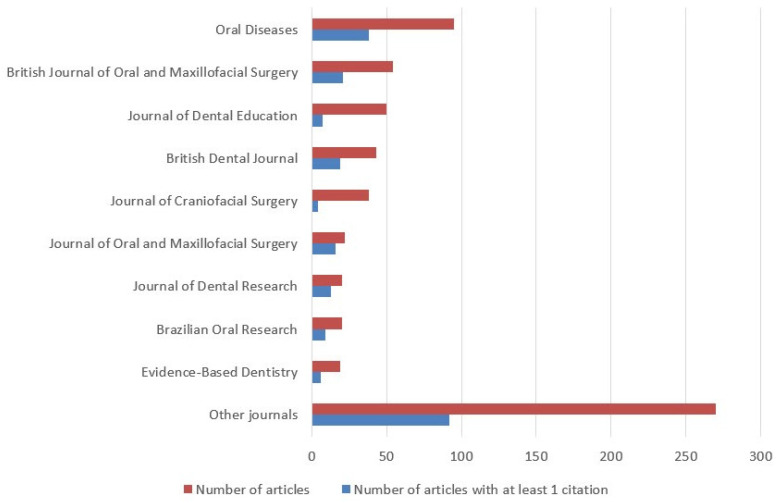
Dental journals with the highest number of COVID-19 articles.

**Figure 3:**
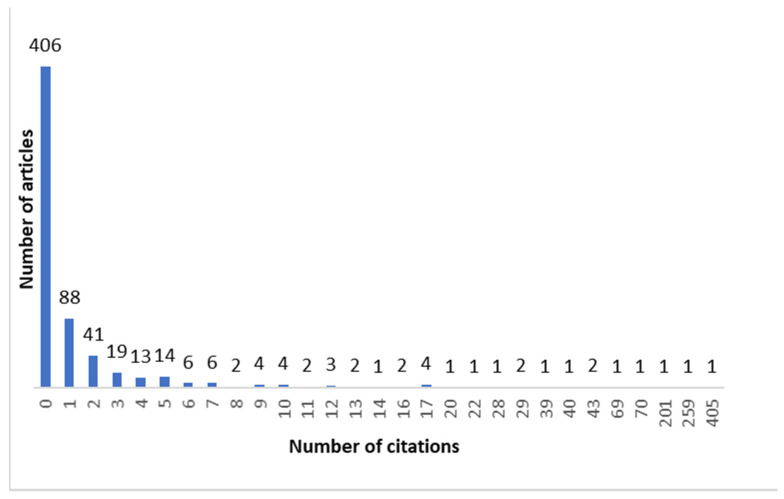
Frequency of PubMed citations.

**Figure 4:**
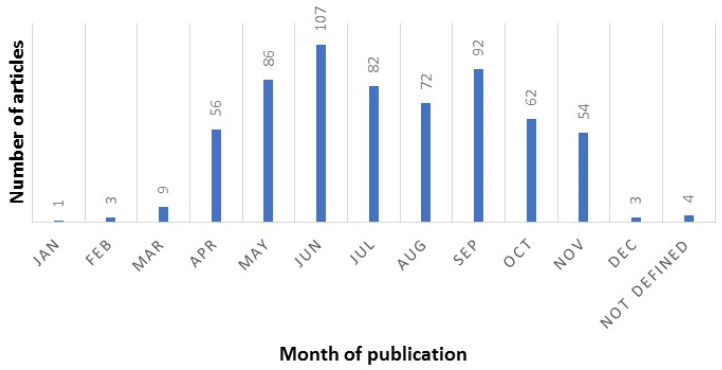
Frequency of articles published in different months.

The search retrieved a total of 659 articles, of which 28 were excluded. A total of 631 articles were included in the analysis (Figure 1). Oral Diseases has published 95 COVID-19 articles (15.1%), followed by the British Journal of Oral and Maxillofacial Surgery with 54 articles (8.6%), the Journal of Dental Education with 50 articles (7.9%), the British Dental Journal with 43 articles (6.8%) and the Journal of Craniofacial Surgery with 38 articles (6.0%). In addition, articles published in the Journal of Oral and Maxillofacial Surgery were most likely to receive at least one citation compared with the other top journals (Figure 2). Most of the articles were from researchers from the United States (168), the United Kingdom (120), Brazil (83), Italy (64), China (53), and India (35). The number of PubMed citations of the published articles ranged from 0 to 405. Of all articles, 406 of them (64.3%) did not have any citations, and only 32 (5.0%) had 10 or more citations (Figure 3). June 2020 had the highest number of published articles with 107 COVID-19 papers, followed by September 2020 with 92 articles (Figure 4).

**Figure 5:**
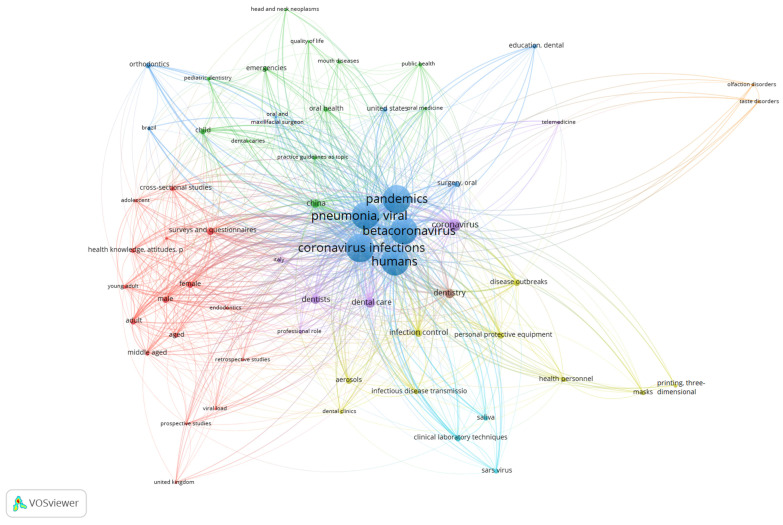
Network visualization of the keywords showing 60 nodes and 8 clusters.

For science mapping analysis, all 659 articles initially retrieved in the PubMed search were included. A total of 60 keywords met the minimum co-occurrence. The most frequently used keywords were coronavirus infections (*n* = 349), pandemics (*n* = 349), humans (*n* = 348), viral pneumonia (*n* = 347), betacoronavirus (343), dental care (*n* = 70), dentistry (*n* = 40), dentists (*n* = 33), China (*n* = 32) and infection control (*n* = 30). In addition, network analysis revealed the categorization of these keywords in eight clusters (Figure 5).

## 4. Discussion

The COVID-19 pandemic caused an infodemic of published articles on topics ranging from the economy, cultural science and arts to engineering and health sciences [7,8,9,10,11,12,13]. Dentistry and dental professionals have been directly affected by and affecting the COVID-19 pandemic in several aspects, including practice management, infection control, dental education, diagnostic methods, and oral manifestations of the disease. In response to these unmatched effects and outcomes, dental journals have been active in the publication and dissemination of information related to COVID-19. The number of COVID-19 publications in dental journals increased from the beginning of 2020, reaching its peak in June. The number of COVID-19 articles published monthly in dental journals has slightly decreased thereafter. However, COVID-19 seems to remain an important topic of discussion in dental journals for the rest of 2020.

Researchers from the United States contributed the highest number of articles. The United States is the country with the second highest number of scientific and technical journal articles [14]. Moreover, all other countries with a high number of COVID-19 articles in dental journals were among the top countries in the world in the number of scientific publications [15]. One interesting finding was that articles from Chinese researchers were most likely to be cited at least once. In fact, the ratio of the number of articles with at least one citation to the number of all articles from that country was the highest for China compared with other top countries (57%). We can speculate that as the first cases of COVID-19 were reported in China, articles from Chinese researchers were most seen and cited in the literature.

The authors observed that the majority of the analyzed articles were not cited even once. This can reflect the weak overall impact of COVID-19 articles in dental journals. Several attributing factors can be responsible in this finding. Many of the articles were commentaries or letters to the editor which show the perspective of an individual or the strategy of an institution facing the COVID-19 crisis. Although reading the generously shared experiences of health care staff all around the world in their battle against COVID-19 is valuable, from the citation standpoint, commentaries generally tend to receive fewer citations compared to original contributions [16]. However, some of the analyzed articles enjoyed a high number of citations. One example was the article titled, “High expression of ACE2 receptor of 2019-nCoV on the epithelial cells of oral mucosa”, published in the International Journal of Oral Science [17]. In this original article, the authors have shown that ACE2, which is reportedly the major host cell receptor of SARS-COV-2 [18,19], is expressed on oral mucosal cells, making the oral cavity a potentially high-risk site for COVID-19 infections. This article has received the highest number of citations among the analyzed articles, with 405 PubMed citations. The second most cited article among the analyzed articles was the article titled, “Transmission routes of 2019-nCoV and controls in dental practice”, published in the International Journal of Oral Science, which has attracted 259 citations [20]. In this review, the authors discuss different transmission routes of COVID-19 in dentistry and present some ways to manage and control these possible pathways. Another article with a high number of citations was published in the Journal of Dental Research, titled “Coronavirus Disease 2019 (COVID-19): Emerging and Future Challenges for Dental and Oral Medicine”, with 201 citations in PubMed [21].

Science mapping analysis showed that, predictably, the hottest keywords in the COVID-19 output of dental journals were the ones corresponding to the terms used for COVID-19 and SARS-CoV-2, as well as general terms such as humans and pandemics. Then, keywords pertaining to dentistry such as dental care, dentistry and dentists were the most used keywords. Other notable keywords included China, which is where the first cases of the disease were reported, along with infection control and personal protective equipment, which are among the most important topics related to dental care in the context of COVID-19.

A wide range of topics was discussed in the analyzed articles, including infection control and the management of practices and patients during the outbreak for dental offices, dental schools or different departments, new approaches for virtual and online dental education, the prospects of dentistry during and after COVID-19, oral manifestations of the disease, the diagnostic potential of saliva, and immunological features of COVID-19 and SARS-COV-2 related to the oral cavity. These topics and their associations are reflected in the keyword visualization using the science mapping approach, showing the wide range of implications of COVID-19 for dentistry. However, as of December 2020, there are more than 80,000 articles in the COVID-19 database of PubMed in total, showing a considerably higher number of articles in biomedical scientific journals compared to dental journals. In addition, while the infodemic of published COVID-19 studies led to several retracted and withdrawn articles [22], there was no retraction of articles published in dental journals in the special web page of retracted COVID-19 papers [23].

The present analysis used a science mapping approach to provide an overview on the output of dental journals regarding COVID-19. Due to different classifications of journals for article types, categorizing the types of analyzed papers was not performed. To our knowledge, this study was the first study attempting to analyze the COVID-19 output of dental journals using a science mapping approach. Further subject-specific reviews are recommended in order to address important issues such as practice management, infection control, dental education, and oral diagnostic approaches. Although several effective vaccines have been developed recently, the global community is still dealing with the ongoing pandemic. Therefore, accurate and reliable scientific information passed through meticulous review filters can give us valuable information on how to deal with the pandemic and its long-lasting outcomes.

## 5. Conclusions

The dental community has been active in the publication of COVID-19 articles from the beginning of the pandemic. A total of 659 articles were found in the PubMed COVID-19 database with a wide range of topics. The papers published by dental journals explore issues such as the management of clinical practices during the outbreak, infection control in the dental setting, signs and symptoms of COVID-19 affecting the oral cavity, and the impact of the COVID-19 pandemic on educational and clinical programs. Further subject-specific reviews are recommended in order to address important implications of COVID-19 in dentistry.

## Data Availability

The data used for this analysis are available at: http://dx.doi.org/10.17632/7bjhtwpfg3.1 (accessed on 1 February 2021).
